# Addendum: Poaceae-specific cell wall-derived oligosaccharides activate plant immunity via OsCERK1 during *Magnaporthe oryzae* infection in rice

**DOI:** 10.1038/s41467-021-24162-0

**Published:** 2021-06-24

**Authors:** Chao Yang, Rui Liu, Jinhuan Pang, Bin Ren, Huanbin Zhou, Gang Wang, Ertao Wang, Jun Liu

**Affiliations:** 1 State Key Laboratory of Plant Genomics, Institute of Microbiology, Chinese Academy of Sciences, Beijing, China; 2 CAS Center for Excellence in Biotic Interactions, University of Chinese Academy of Sciences, Beijing, China; 3 State Key Laboratory for Biology of Plant Diseases and Insect Pests, Institute of Plant Protection, Chinese Academy of Agricultural Sciences, Beijing, China; 4 National key Laboratory of Plant Molecular Genetics, Center for Excellence in Molecular Plant Sciences, Institute of Plant Physiology and Ecology, Chinese Academy of Sciences, Shanghai, China

**Keywords:** Effectors in plant pathology, Plant signalling

Correction to: *Nature Communications* 10.1038/s41467-021-22456-x, published online 12 April 2021

During the peer review of this article, a related work demonstrating that 3^1^-β-D-Cellobiosyl-glucose and 3^1^-β-D-Cellotriosyl-glucose could elicit immune responses in Arabidopsis and other dicots was published by Rebaque et al.^1^

This work also showed that *Arabidopsis thaliana* was exposed to mixed-link glucans during interaction with the oomycete *Hyaloperonospora arabidopsidis*, demonstrating that in addition to being released from poaceae cell walls, such oligosaccharides could also be derived from microbes.
