# Supplementary material for: Poaceae-specific cell wall-derived oligosaccharides activate plant immunity via OsCERK1 during Magnaporthe oryzae infection in rice
Source: Nat Commun. 2021 Apr 12;12:2178. doi: 10.1038/s41467-021-22456-x (PMC8042013; doi:10.1038/s41467-021-22456-x)
Supplement: Supplementary file 1 — Supplementary Information [file 41467_2021_22456_MOESM1_ESM.pdf]

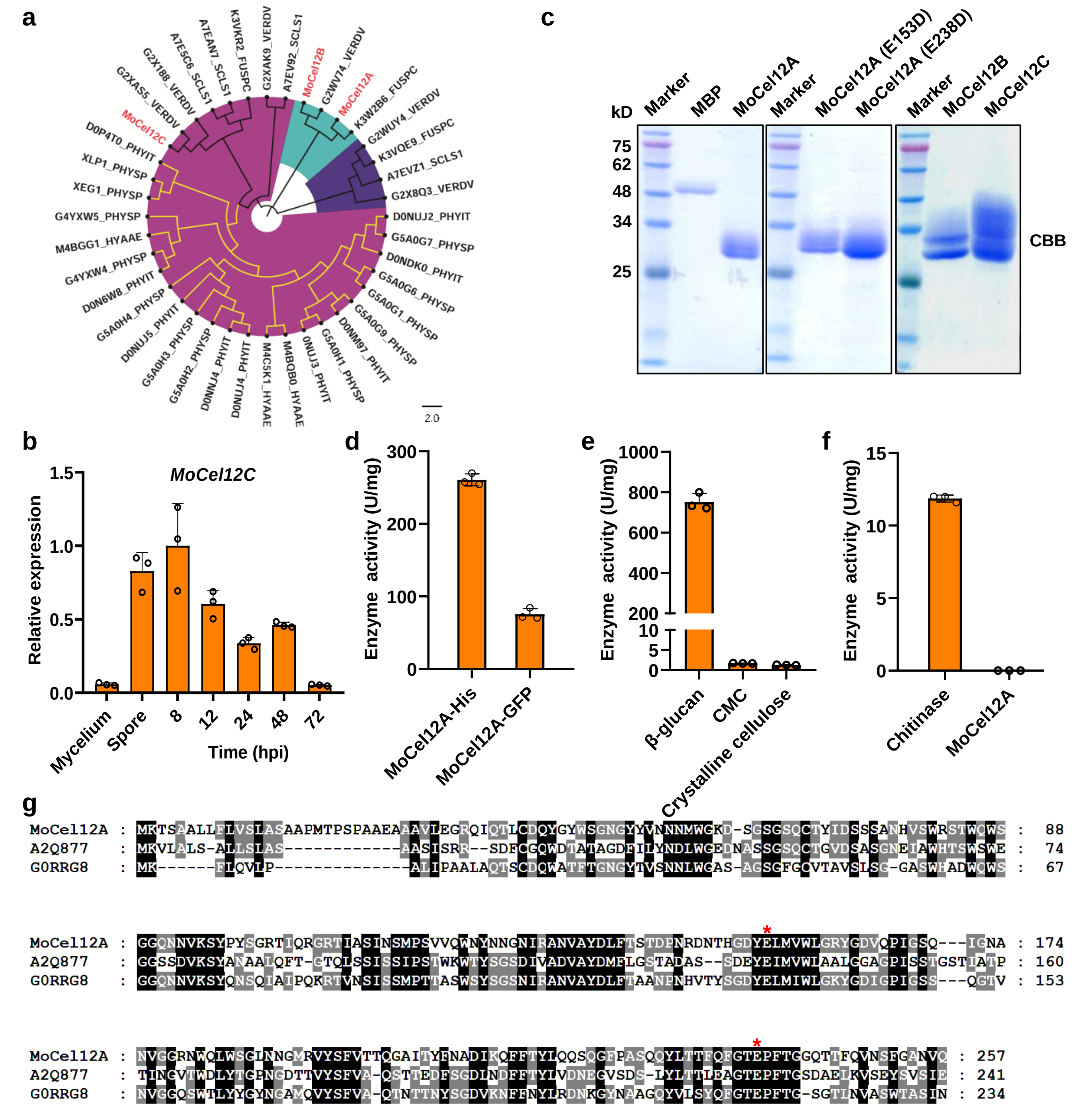

Supplementary Fig. 1 MoCel12A/B prefer β-glucan as the substrate.

(**a**) Phylogenetic tree of MoCel12A homolog proteins. The phylogenetic tree was generated using MEGA 7.0. The scale bar represents 20% weighted sequence divergence. PHYSP, *Phytophthora sojae*; PHYIT, *Phytophthora infestans*; *HYAAE, Hyaloperonospora arabidopsidis*; VERDV, *Verticillium dahlia*; SCLS1, *Sclerotinia sclerotiorum*; FUSPC, *Fusarium pseudograminearum.*

(**b**) *MoCel12C* transcription levels in different infection stages in *M. oryzae.* The fungi were grown in CM media, and the mycelium and spores were collected for RT-qPCR assays. The rice leaves were inoculated with *M. oryzae* spores at a concentration of 1 x 10^5 per ml. The leaves were sampled at indicated time points for RT-qPCR assays. The *M. oryzae MoActin* was used as a reference gene. hpi, hours post inoculation. Values are means ± SD (n = 3 biological replicates).

(**c**) Purification of MoCel12 proteins from *Pichia pastoris*. The purified His-tagged MoCel12A, MoCel12A (E153D), MoCel12A(E238D), MoCel12B, and MoCel12C were stained by coomassie brilliant blue in SDS-PAGE gel.

(**d**) The enzymatic activity of recombinant MoCel12A purified from *P. pastoris* and *M. oryzae*. The recombinant MoCel12A-His and MoCel12A-GFP were expressed and purified from *P. pastoris* and *M. oryzae*, respectively. The hydrolytic activity of these proteins was examined using barley β-glucan as the substrate. Values are means ± SD (n = 3 biological replicates).

(**e**) The hydrolytic activity of MoCel12A on carboxymethyl-cellulose (CMC) and crystalline cellulose. The enzymatic activity assays are as in (d).

(**f**) MoCel12A does not hydrolyze chitin. The enzymatic assays were performed using chitin as the substrate. The chitinase from *Streptomyces griseus* served as a positive control. Others are as in (d).

(**g**) The conserved amino acid residues of several MoCel12A homolog proteins from *Aspergillus niger* (A2Q877) and *Trichoderma reesei* (G0RRG8). The asterisk indicates the amino acid residue essential for hydrolytic activity.

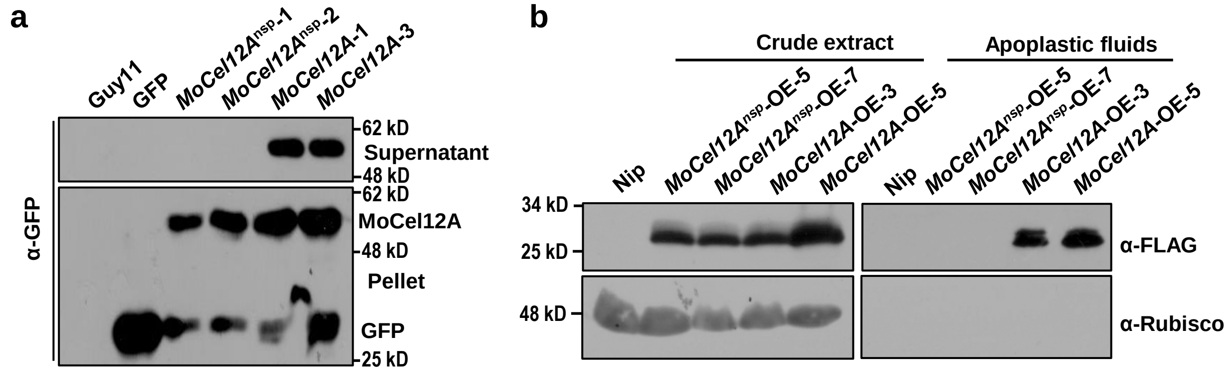

Supplementary Fig. 2 MoCel12A is a secreted protein.

(**a**) MoCel12A is secreted by *M. oryzae* into the media. MoCel12A and MoCel12A^{nsp} were fused with GFP and expressed in *M. oryzae*. The proteins were examined in the supernatant and the pellet with anti-GFP antiserum. MoCel12A^{nsp} is a truncated protein without secreting signal.

(**b**) MoCel12A is localized to the rice apoplast. The proteins were fused with FLAG epitope at C-terminal. The rice apoplastic fluids of two-week-old WT, *MoCel12A^{nsp}*-OE, and *MoCel12A*-OE plants were extracted via vacuum extraction. The FLAG-fused MoCel12A^{nsp} and MoCel12A proteins were probed by anti-FLAG antibody. Rice leaf crude extracts were probed by anti-Rubisco to evaluate the contamination from cytosolic proteins.

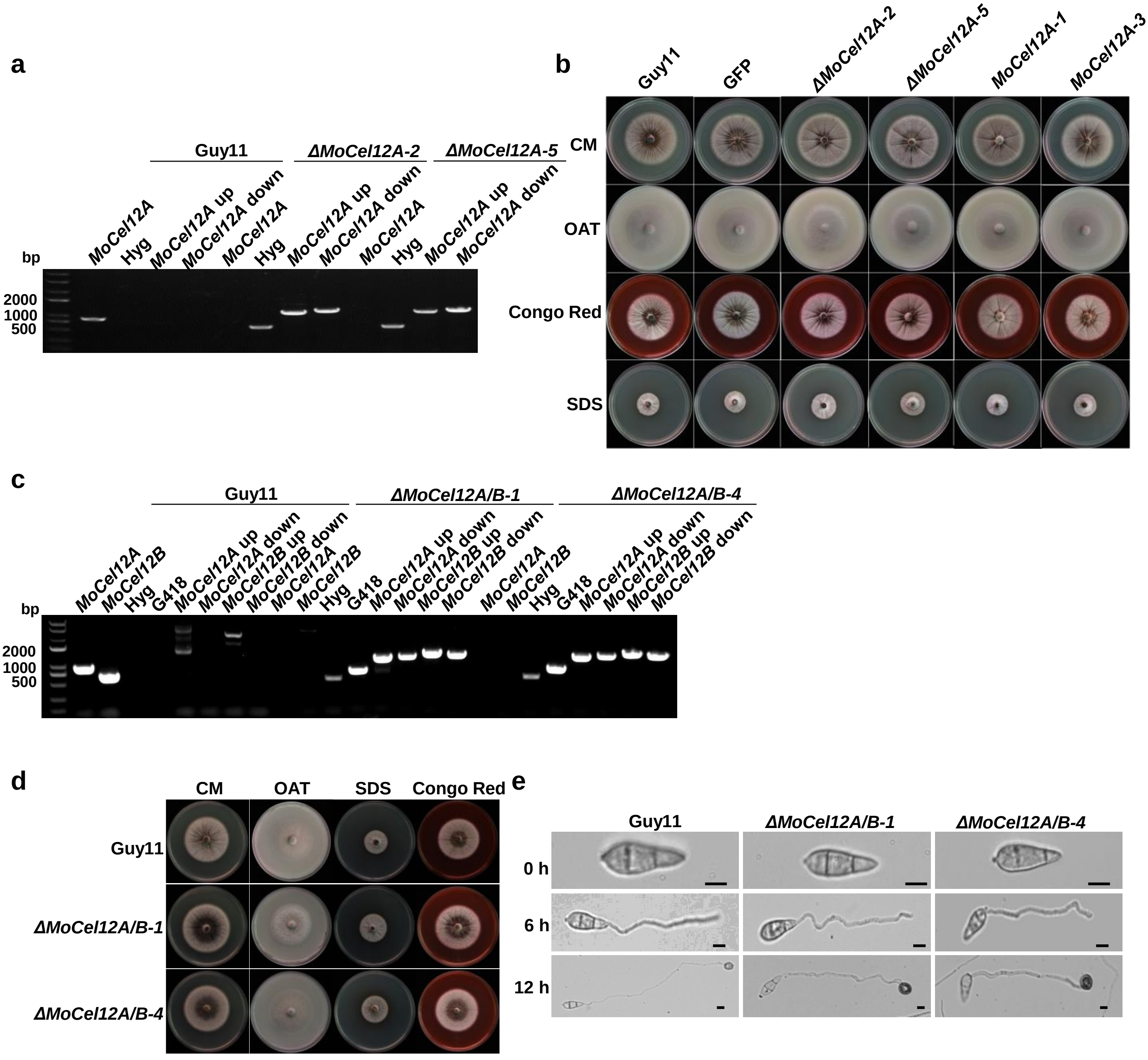

Supplementary Fig. 3 Knocking out *MoCel12A* and *MoCel12B* did not affect the growth of *M. oryzae.*

(**a**) PCR verification of the *MoCel12A* knockout strains. The upstream and downstream genomic sequences of *MoCel12A* were amplified with primers listed in Supplementary Table 2 and were inserted into the positions flanking the hygromycin-resistance cassette of the pGKO-HPT vector to generate gene-knockout plasmids. The wild-type strain Guy11 and two independent *△MoCel12A* mutants were analyzed using PCR with the primers of *MoCel12A*, hygromycin-specific primers *Hyg*, and *MoCel12A* upstream and downstream sequence.

(**b**) The morphology of the Guy11, *△MoCel12A* mutant, and *MoCel12A*-overexpressing strains (*MoCel12A-1* and *-3*) on different media. The mycelia were grown on CM, OAT or the media containing 300 µg/ml Congo Red, and 0.01% SDS for 7 d at 28℃. GFP-overexpressing strain (GFP) served as a negative control.

(**c**) PCR verification of the *MoCel12A* and *MoCel12B* double-mutant strains. The hygromycin- and G418-resistance cassette of the pGKO-HPT vector was used to generate gene-knockout plasmids. Others are as in (a).

(**d**) The morphology of Guy11 and *△MoCel12A/B* mutants on different media. *ΔMoCel12A/B-1* and *ΔMoCel12A/B-4* are two independent double knockout strains. Others are as in (b).

(**e**) Germination of conidial spores of the WT and *MoCel12A/B* mutant. Conidia were germinated on glass cover slips. The germination tubes and appressorium development were observed at the indicated time points. Bar = 10 µm.

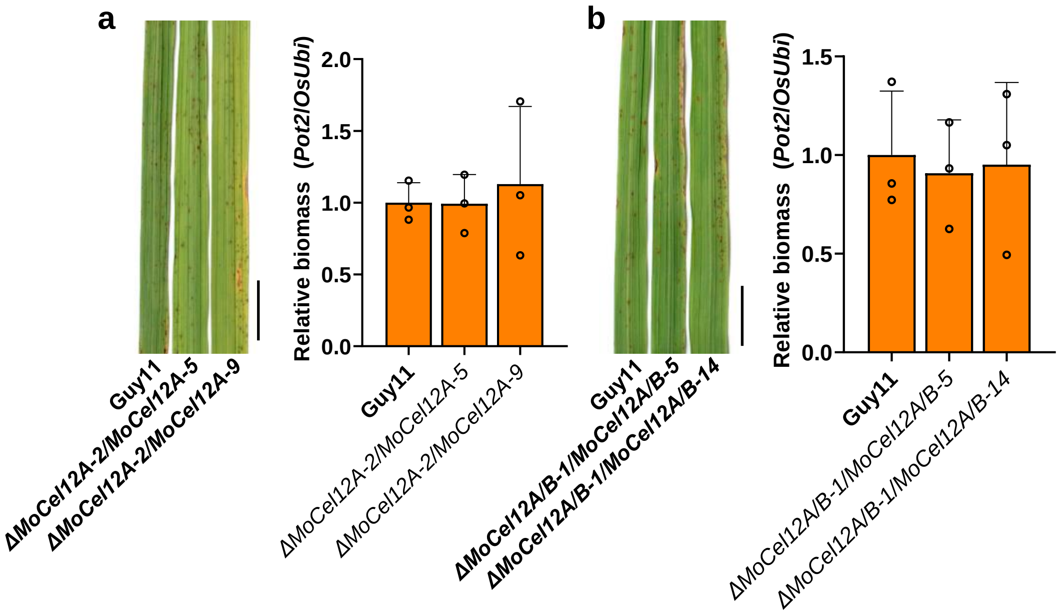

Supplementary Fig. 4 Disease symptoms of rice leaves infected with the complementation strains of *ΔMoCel12A* and *ΔMoCel12A/B*.

(**a**) Disease symptoms of rice leaves infected with Guy11 and the complementation strains of *ΔMoCel12A-2*. The *ΔMoCel12A-2* strain was complemented with the native promoter driven *MoCel12A*. -5 and -9 are two independent transformants. Conidial suspensions (1×10^5 conidia per ml in 0.02% Tween-20) were sprayed onto the leaf surfaces of 4-week-old seedlings. Images were taken at 5 dpi. Bar = 1cm. Right panel, the relative fungal biomass determined by qPCR. Values are means ± SD (*n* = 3 biological replicates).

(**b**) Disease symptoms of rice leaves infected with Guy11 and the complementation strains of *ΔMoCel12A/B-1*. The *ΔMoCel12A/B-1* strain was complemented with their native promoters driven *MoCel12A* and *MoCel12B.* Others are as in (a).

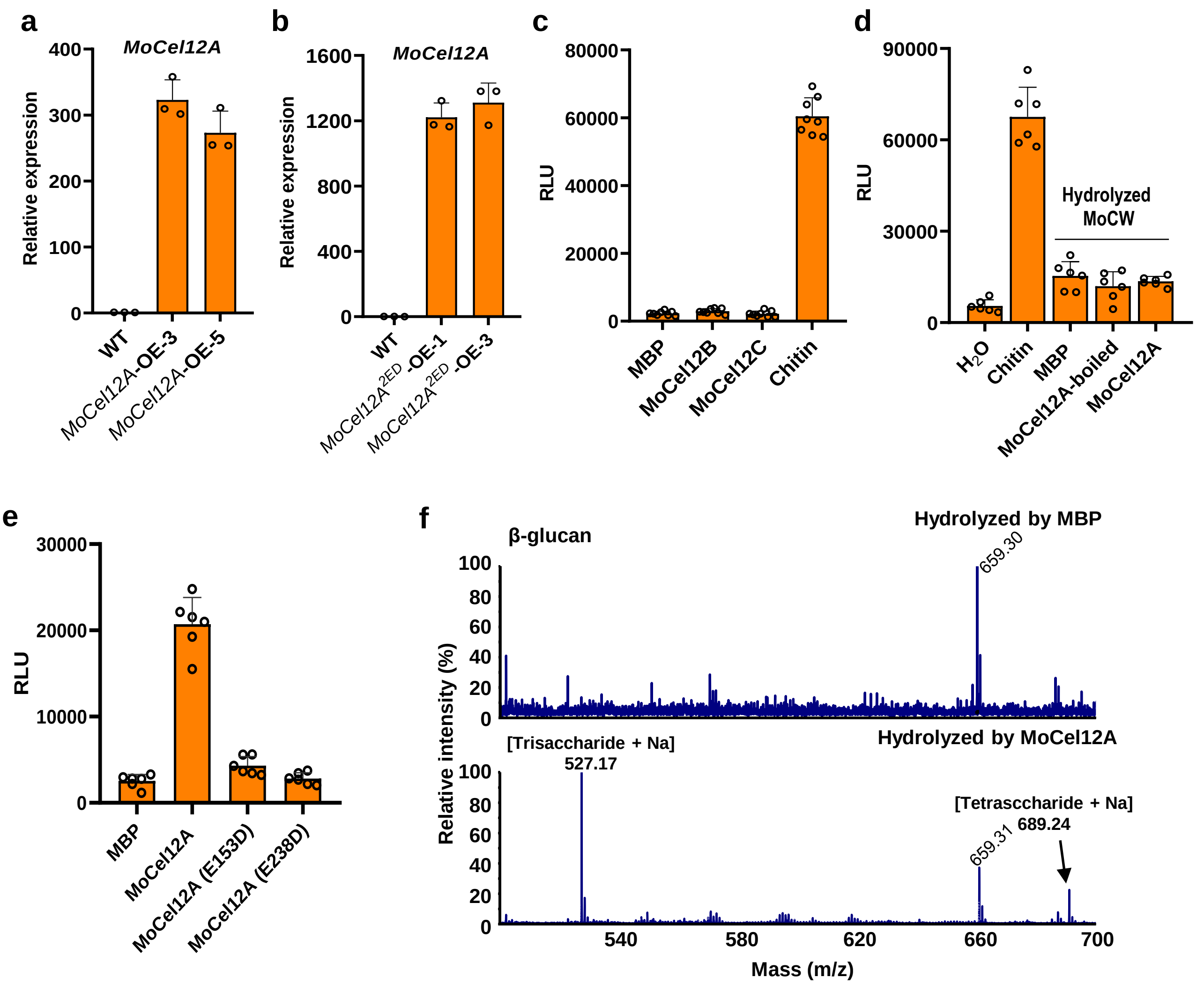

Supplementary Fig.5 MoCel12A hydrolyzed β-glucan to activate immune response in rice cells.

(**a**) *MoCel12A* and (**b**) *MoCel12A^{2ED}* expression levels in the transgenic plants. The leaves were sampled from two-week-old rice seedlings of the *MoCel12A* and *MoCel12A^{2ED}* overexpressing lines and the expression of *MoCel12A* was examined by RT-qPCR. Values are means ± SD (n = 3 biological replicates).

(**c**) MoCel12B and MoCel12C cannot activate ROS burst in rice suspension cells. Recombinant His-tag proteins were purified from *Pichia pastoris* and 1 μg/ml protein each was used for the L012-based ROS burst assays. Chitin (1 μg/ml) served as a positive control. MBP served as a negative control. Values are means ± SD (n = 8 biological replicates). RLU, relative light units.

(**d**) MoCel12A-digested *M. oryzae* cell wall (MoCW) cannot enhance ROS burst in rice. Aliquots (1 ml) of a 50 mg/ml suspension of isolated MoCW in 50 mM sodium acetate, pH 5.5, were incubated for 2 h at 37℃ with 5 μg MoCel12A. MBP and boiled MoCel12A served as the negative controls. Chitin (1 μg/ml) served as a positive control. After incubation, the supernatants (1/100, v/v) were used for the ROS burst assays. Values are means ± SD (n = 6 biological replicates).

(**e**) MoCel12A-hydrolyzed β-glucan activated the ROS burst in rice. Aliquots (1 ml) of β-glucan (8 mg/ml) in 50 mM sodium acetate, pH 5.5, were incubated with 5 μg purified recombinant MBP-His, MoCel12A-His, MoCel12A(E153D)-His, and MoCel12A(E238D)-His for 2 h at 37℃, respectively. After incubation, the filtered supernatants (1/100, v/v) were used for the ROS burst assay. Values are means ± SD (n = 6 biological replicates).

(**f**)The oligosaccharides of MoCel12A hydrolyzed β-glucan detected by MALDI-TOF analysis. The filtered supernatants were used for the analysis, using 10 mg/ml 2,5-dihydroxybenzoic acid in 2:1 acetonitrile:water as the matrix. All indicated peaks are single charged ions of oligosaccharides on the reducing end as Na^+ using 3-aminoquinoline (3-AMQ) as ionic liquid matrix.

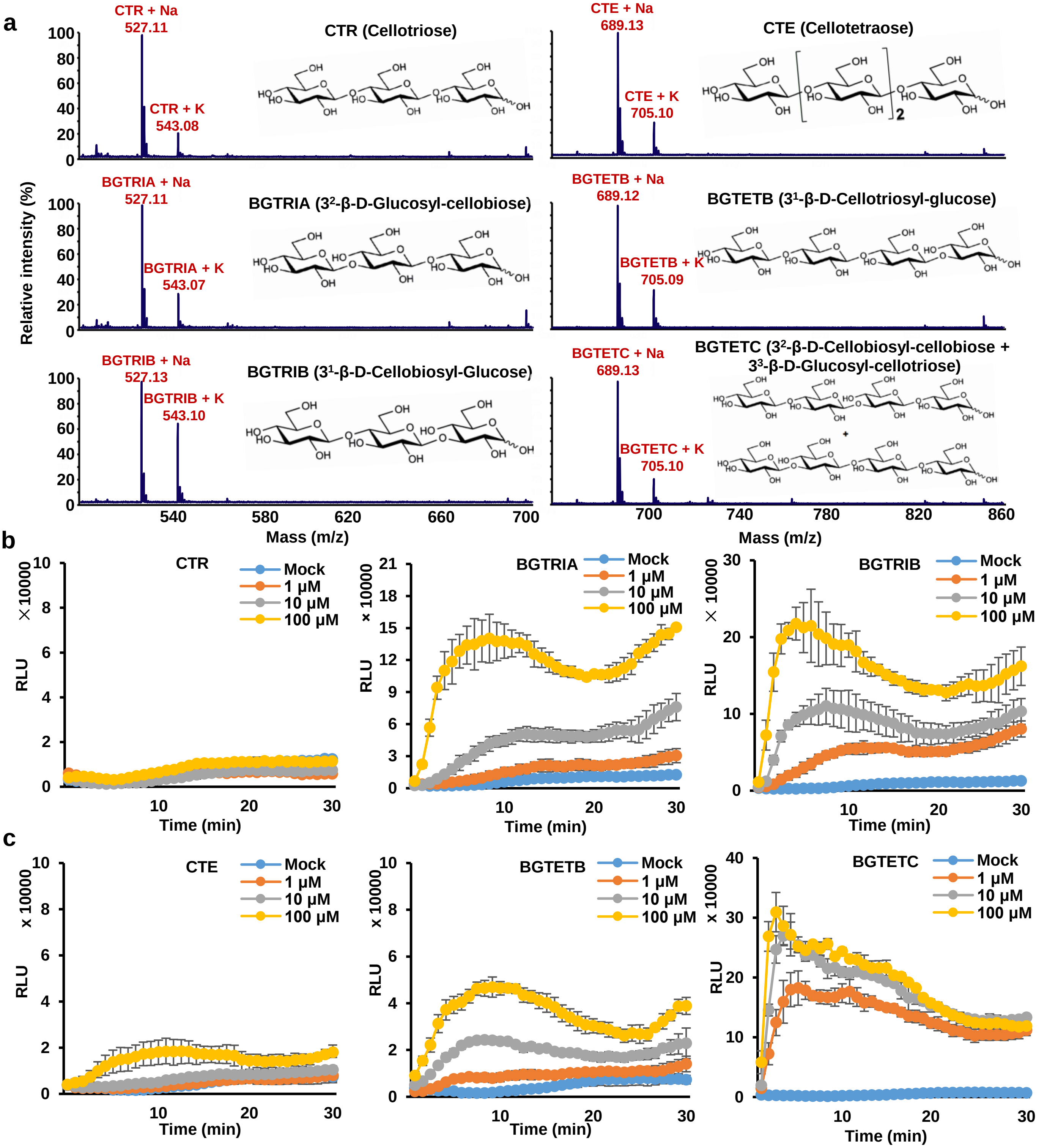

Supplementary Fig. 6 The specific oligosaccharides activate ROS burst in a dose-dependent manner.

(**a**) The tested trisaccharides and tetrasaccharides were verified by MALDI-TOF analysis. All indicated peaks are single charged ions of oligosaccharides on the reducing end with Na^+ using 3-aminoquinoline (3-AMQ) as ionic liquid matrix.

(**b**) The trisaccharides activate ROS burst in a dose-dependent manner. Different concentrations of CTR (Cellotriose), BGTRIA (3^2-β-D-Glucosyl-cellobiose), and BGTRIB (3^1-β-D-Cellobiosyl-Glucose) were used for the L012-based ROS burst assay. Values are means ± SD (*n* = 4 biological replicates). RLU, relative light units.

(**c**) The tetrasaccharides activate the ROS burst in a dose-dependent manner. Different concentrations of CTE (Cellotetraose), BGTETB (3^1-β-D-Cellotriosyl-glucose), and BGTETC (3^2-β-D-Cellobiosyl-cellobiose + 3^3-β-D-Glucosyl-cellotriose) were used for the ROS burst assays. Others are as in (b).

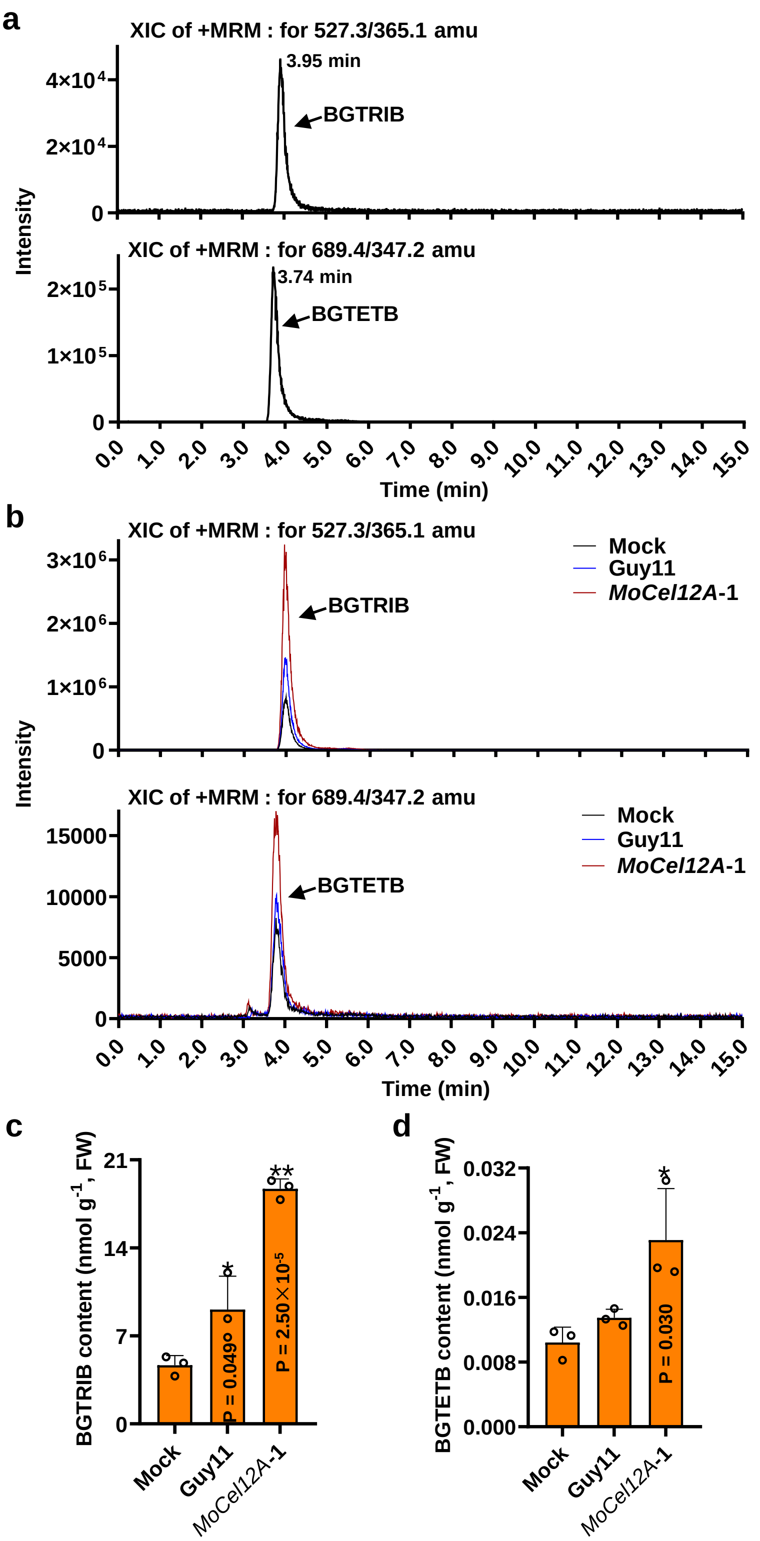

Supplementary Fig. 7 ***M. oryzae*** **infection enhanced the oligosaccharides content in rice apoplastic fluids**.

(**a**) Multiple reaction monitoring (MRM) chromatograms for BGTRIB and BGTETB. An aliquot (1 μl) of BGTRIB and BGTETB (0.8 μM each) was injected onto a RP-HPLC column, with the eluate monitored using ESI-MS/MS. 527.3/365.1 and 689.4/347.2 are the parental and daughter M/Z for BGTRIB and BGTETB, respectively.

(**b**) MRM chromatograms for the BGTRIB and BGTETB in apoplastic fluids of Guy11 and *MoCel12A* overexpression strain-infected rice leaves. Conidial suspensions of Guy11 and *MoCel12A*-1 (5×10^5 conidia per ml in 0.02% Tween-20) were sprayed onto the leaf surfaces of three-week-old rice seedlings. The leaves were harvested for the apoplastic fluid isolation at 3 dpi. Experiments were performed with three independent biological replicates.

(**c**) BGTRIB and (**d**) BGTETB content in apoplastic fluids of Guy11 and *MoCel12A* overexpression strain-infected rice leaves. * and ** indicate significant differences from the treatment of Mock at $P < 0.05$ and 0.01, respectively (Two-sided Student's *t*-test). FW, fresh weight.

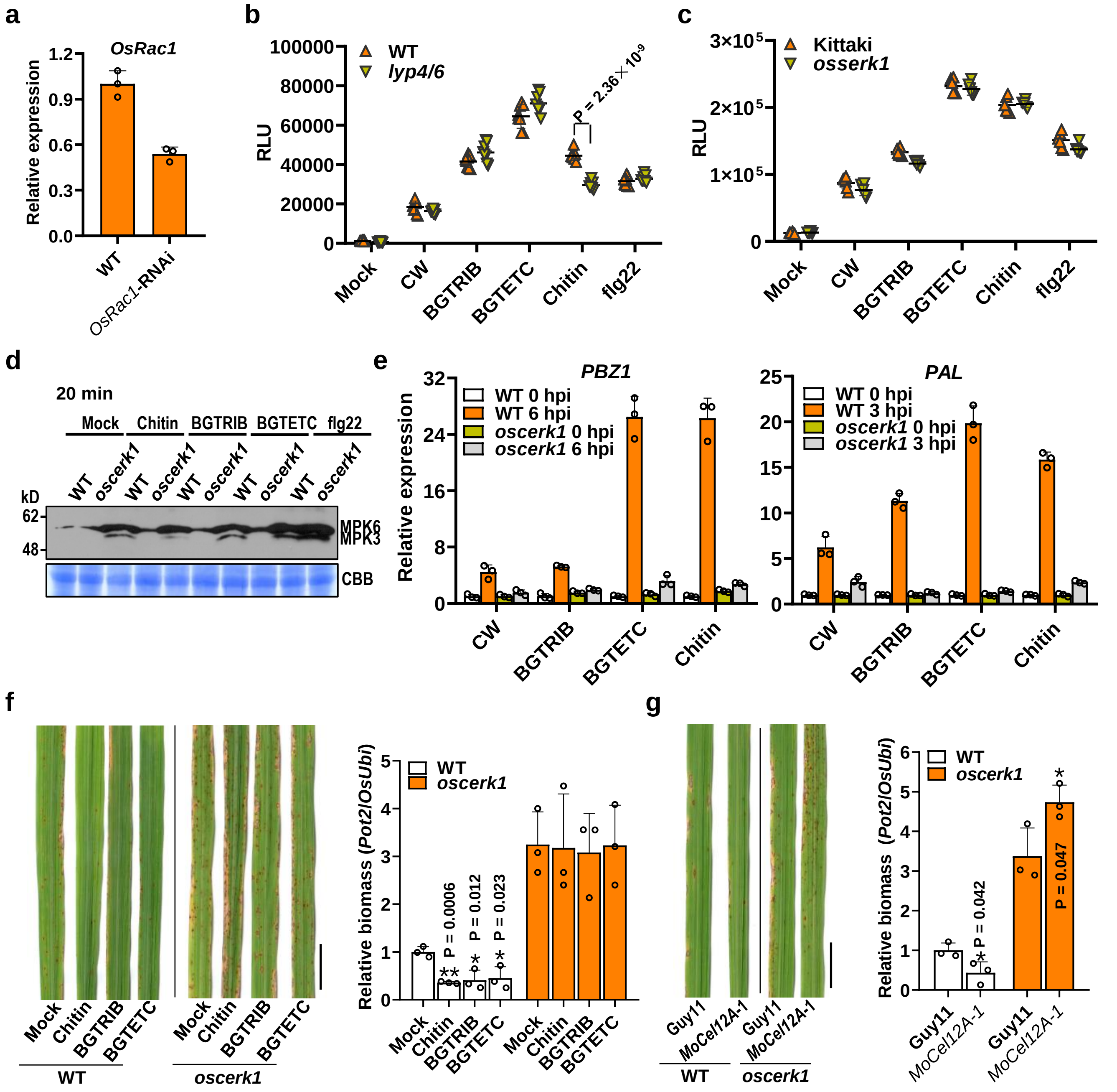

Supplementary Fig. 8 OsCERK1 specifically perceives MoCel12A-released trisaccharides and tetrasaccharides from rice cell walls.

(**a**) *OsRac1* expression levels in the *OsRac1*-RNAi plants. The leaves were sampled from two-week-old rice seedlings of the *OsRac1*-RNAi plants and the expression of *OsRac1* was examined by RT-qPCR. Values are means ± SD (n = 3 biological replicates).

(**b**) ROS production induced by oligosaccharides in WT and *lyp4/6* mutant. Sterile water (Mock), filtered supernatants (1/100, v/v) of MoCel12A-hydrolyzed rice cell walls (CW), 10 mM BGTRIB, 10 mM BGTETC, and 5 μg/ml chitin were used for ROS burst assays.100 nM flg22 served as a positive control. Values are means ± SD (n = 8 biological replicates).

(**c**) ROS production induced by oligosaccharides in WT and *osserk1* mutant. Others are as in (b). Values are means ± SD (n = 6 biological replicates).

(**d**) The BGTRIB and BGTETC induced MAP kinase activation was compromised in *oscerk1* suspension cells. Chitin (5 μg/ml), BGTRIB, and BGTETC (10 μM) were used for the assays. flg22 (100 nM) served as the positive control.

(**e**) BGTRIB and BGTETC induced PTI marker gene expression was suppressed in *oscerk1* mutant. The samples were collected for RT-qPCR assays at the indicated time points. Values are means ± SD (n = 3 biological replicates).

(**f**) Pre-treatment with BGTRIB and BGTETC on WT and *oscerk1* plants. Four-week-old rice plants were treated with 50 μg/ml chitin, 100 μM BGTRIB or BGTETC for 3 h, then the leaves were spray-inoculated with conidial suspensions (1×10^5 conidia per ml in 0.02% Tween-20) for 5 d. Bar = 1 cm. The right panel is the relative fungal biomass. Values are means ± SD (n = 3 biological replicates). * indicates significant differences from the Mock at $P < 0.05$ (Two-sided Student's t-test).

(**g**) Disease symptoms of rice leaves infected with the Guy11 and *MoCel12A*-overexpression strains in the *oscerk1* plants. Others are as in (f).

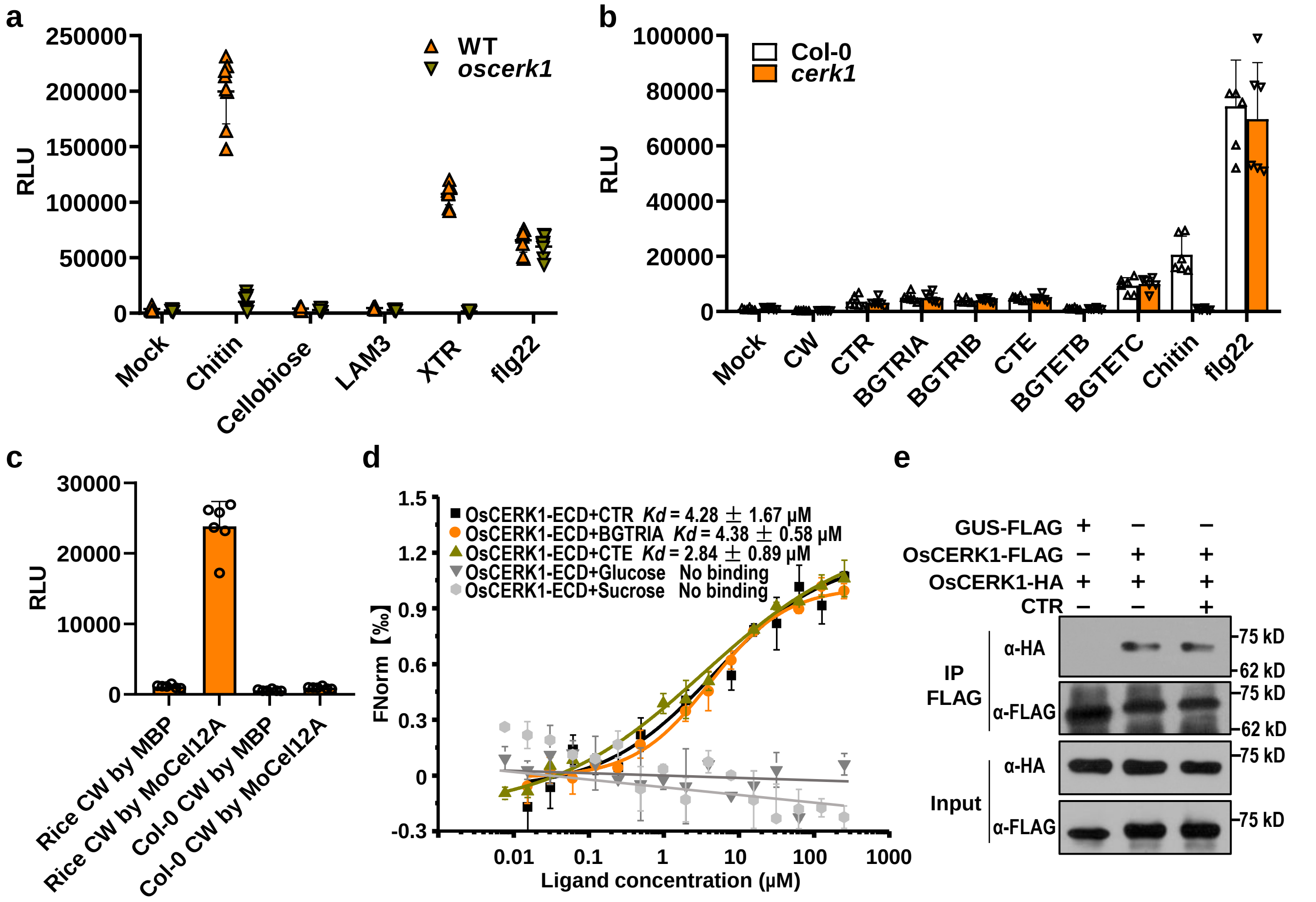

Supplementary Fig. 9 OsCERK1 perceives specific oligosaccharides.

(**a**) Xylotriose can activate ROS burst in rice suspension cells. 10 μM each of cellobiose, laminaritriose (LAM3), and xylotriose (XTR) were used for the ROS burst assays. Chitin (2 μg/ml) and flg22 (100 nM) served as the positive controls. Values are means ± SD (*n* = 8 biological replicates).

(**b**) ROS production induced by the specific oligosaccharides in Col-0 and *cerk1* mutant leaves. Two-week-old Col-0 and *cerk1* seedlings were treated with sterile water (Mock), filtered supernatants (1/100, v/v) of MoCel12A-hydrolyzed rice cell walls (CW), 100 μM oligosaccharides, and 100 μg/ml chitin for ROS burst assays.100 nM flg22 served as a positive control. Values are means ± SD (*n* = 6 biological replicates).

(**c**) MoCel12A-hydrolyzed *Arabidopsis* cell wall cannot activate the ROS burst in rice suspension cells. Aliquots (1 ml) of a 50 mg/ml suspension of isolated Col-0 and rice cell wall in 50 mM sodium acetate, pH 5.5, were incubated 2 h at 37℃ with 5 μg MBP and MoCel12A, respectively. After incubation, the supernatants (1/100, v/v) were used for the ROS burst assay. CW, cell wall. Values are means ± SD (*n* = 6 biological replicates).

(**d**) MST assays show the binding of OsCERK1-ECD to CTR, BGTRIA, and CTE, respectively. The recombinant OsCERK1-ECD was incubated in NT standard capillaries. The solid curve is the fit of the data points to the standard K_d-fit function. K_d, dissociation constant. Bars represent ± SD (*n* = 3 biological repeats).

(**e**) CTR cannot trigger OsCERK1 dimerization in rice. FLAG- and HA-tagged OsCERK1 were co-expressed in WT rice protoplasts. GUS-FLAG served as the negative control. The protoplasts were treated with 10 μM CTR for 15 min. Co-immunoprecipitation was performed using anti-FLAG antibody and subjected to immunoblot analysis with anti-FLAG or anti-HA antibody as indicated. The experiment was repeated three times with similar results.

Supplementary Table 1. Primers used for this study

Primers name	Primers sequence (5'→3')	Description
PAL-F	TGAATAACAGTGGAGTGTGGAG	qRT-PCR
PAL-R	AACCTGCCACTCGTACCAAG	qRT-PCR
PBZ1-F	GGTGTGGGAAGCACATACAA	qRT-PCR
PBZ1-R	GTCTCCGTCGAGTGTGACTTG	qRT-PCR
Pot2-F	ACGACCCGTCTTTACTTATTTGG	qRT-PCR
Pot2-R	AAGTAGCGTTGGTTTTGTTGGAT	qRT-PCR
OsActin-F	TTATGGTTGGGATGGGACA	qRT-PCR
OsActin-R	AGCACGGCTTGAATAGCG	qRT-PCR
OsUbi-F	TTCTGGTCCTTCCACTTTCAG	qRT-PCR
OsUbi-R	ACGATTGATTTAACCAGTCCATGA	qRT-PCR
OsPR3-F	GTCACCGAGGCGTTCTTCA	qRT-PCR
OsPR3-R	GCTTGGAGTCGTCGTTGGT	qRT-PCR
OsPR10-F	CCTCAGCCATGCCATTCAG	qRT-PCR
OsPR10-R	CTTGTCCACGTCCAGGAACTC	qRT-PCR
OsRbohA-F	GAGCGCGTCTGCCAATAAAC	qRT-PCR
OsRbohA-R	TCAATGTAGCCGAGCCCTTC	qRT-PCR
OsRbohD-F	CTGCCGTTTATAGATGGAGGAA	qRT-PCR
OsRbohD-R	GATCCTAAACAAGGCCACTGAC	qRT-PCR
MoCel12A-F	CCTTACTACCTTCCAATTCGGC	qRT-PCR
MoCel12A-R	ACCTTACTGGACGTTGGCTC	qRT-PCR
MoCel12B-F	CATCTGGCCCATCACGGAAT	qRT-PCR
MoCel12B-R	ACGAGTTGTACGATGTCCCG	qRT-PCR
MoCel12C-F	TACCCCAACGCGGTTGTATC	qRT-PCR
MoCel12C-R	CATACTCTGGCGTCGTACCG	qRT-PCR
MoActin-F	CCATGTACCCTGGTCTTTCG	qRT-PCR
MoActin-R	TTCGAGATCCACATCTGCTG	qRT-PCR
OsCERK1(ECD)-pFast-F	CTTTGCGGCGGATCTGCAGAGCGCCGGGTGCGACCTCGCG	Protein expression
OsCERK1(ECD)-pFast-R	TAGTGGTGGTGATGGTGATGAGCTCCTGCAGAAGCTC	Protein expression
CEBiP(ECD)-pFast-F	CTTTGCGGCGGATCTGCAGGCCAACTTCACCTGCGC	Protein expression
CEBiP(ECD)-pFast-R	TAGTGGTGGTGATGGTGATGAGCGAACTGCGACCTCC	Protein expression
MoCel12A pPICZα-F	GAGGCTGAAGCCATGGAATTC ATGGCGCCGATGACACCAT	Protein expression
MoCel12A- pPICZα-R	GGCGGCCGCCGCGGTCTCGAG CTGGACGTTGGCTCCGAAC	Protein expression
MoCel12B- pPICZα-F	GAGGCTGAAGCCATGGAATTC ATGTCCGTGCTGCCGACCA	Protein expression
MoCel12B- pPICZα-R	GGCGGCCGCCGCGGTCTCGAG AACCTGAACCTCCGCCTG	Protein expression
MoCel12C-pPICZα-F	GAGGCTGAAGCCATGGAATTC ATGGTGCCTCACCCCGTC	Protein expression
MoCel12C-pPICZα-R	GGCGGCCGCCGCGGTCTCGAGTGAGCACTGAGAGTACCAC	Protein expression
OsCERK1-Flag/HA-F	GGGGACGAGCTCGGTACCATGGAAGCTTCCACCTCCCT	Transient expression in rice protoplast
OsCERK1-Flag-R	GTCTTTGTAGTCTTCGAATCTCCCGGACATTAGGTTGA	Transient expression in rice protoplast
OsCERK1-HA-R	CATCGTATGGGTAGTCGACTCTCCCGGACATTAGGTTGA	Transient expression in rice protoplast
OsCEBiP-HA-F	GGGGGACGAGCTCGGTACCATGGCCGCGTGCTCCAGGG	Transient expression in rice protoplast
OsCEBiP-HA-R	CATCGTATGGGTAGTCGACAAGGAAACAGATAATGATCAA	Transient expression in rice protoplast

MoCel12A-1390-F	GTTACTTCTGCACTAGGTACCATGAAGACCTCCGCAGCG	Overexpression in rice
MoCel12A-1390-R	TCTTAGAATTCCCGGGGATCCCTGGACGTTGGCTCCGAAC	Overexpression in rice
MoCel12A(nsp)-1390-F	GTTACTTCTGCACTAGGTACCATGGCGCCGATGACACCAT	Overexpression in rice
MoCel12A(nsp)-1390-F	TCTTAGAATTCCCGGGGATCCCTGGACGTTGGCTCCGAAC	Overexpression in rice
HYG-G	AACTCACCGCGACGTCTGTC	Check for *M.oryzae* konockout mutant
HYG-R	CAGAAGAGGATGTTGGCGACC	Check for *M.oryzae* konockout mutant
G418-F	ATGATTGAACAAGATGGATTGCACG	Check for *M.oryzae* konockout mutant
G418-R	TCAGAAGAACTCGTCAAGAAGGC	Check for *M.oryzae* konockout mutant
MoCel12A-UP-F	ATGGTTTTTGCTATAAATGCTTTTAA	Knockout in *M.oryzae*
MoCel12A-UP-R	GGGGAATACCTAGGTCTACA	Knockout in *M.oryzae*
MoCel12A-Dn-F	AAGGTTTCATGAAGATGCTCCG	Knockout in *M.oryzae*
MoCel12A-Dn-R	TTTGAGCTCTGACACATTTCCCAGACC	Knockout in *M.oryzae*
MoCel12B-UP-F	CTCAAAAGCAGTCATGGGG	Knockout in *M.oryzae*
MoCel12B-UP-R	GGGTACTCTCCCCGCGAA	Knockout in *M.oryzae*
MoCel12B-Dn-F	GTTTTGGCCTAACCAACAAAAAC	Knockout in *M.oryzae*
MoCel12B-Dn-R	GCTGGGTCTAGAAAGGTAACG	Knockout in *M.oryzae*
