# Supplementary material for: Poaceae-specific cell wall-derived oligosaccharides activate plant immunity via OsCERK1 during Magnaporthe oryzae infection in rice
Source: Nat Commun. 2021 Apr 12;12:2178. doi: 10.1038/s41467-021-22456-x (PMC8042013; doi:10.1038/s41467-021-22456-x)
Supplement: Supplementary file 3 — Descriptions of Additional Supplementary Files [file 41467_2021_22456_MOESM3_ESM.pdf]

Descriptions of Additional Supplementary Files

Supplementary Data 1

Description: The transcription of M. oryzae GH-family genes during infection on rice leaves
