# Supplementary material for: Poaceae-specific cell wall-derived oligosaccharides activate plant immunity via OsCERK1 during Magnaporthe oryzae infection in rice
Source: Nat Commun. 2021 Apr 12;12:2178. doi: 10.1038/s41467-021-22456-x (PMC8042013; doi:10.1038/s41467-021-22456-x)
Supplement: Supplementary file 5 — Reporting-summary [file 41467_2021_22456_MOESM5_ESM.pdf]

nature research

Corresponding author(s): Jun Liu

Last updated by author(s): Mar 7, 2021

Reporting Summary

Nature Research wishes to improve the reproducibility of the work that we publish. This form provides structure for consistency and transparency in reporting. For further information on Nature Research policies, see our Editorial Policies and the Editorial Policy Checklist.

Statistics

For all statistical analyses, confirm that the following items are present in the figure legend, table legend, main text, or Methods section.

n/a	Confirmed	
☐	☒	The exact sample size (*n*) for each experimental group/condition, given as a discrete number and unit of measurement
☐	☒	A statement on whether measurements were taken from distinct samples or whether the same sample was measured repeatedly
☐	☒	The statistical test(s) used AND whether they are one- or two-sided<br>*Only common tests should be described solely by name; describe more complex techniques in the Methods section.*
☒	☐	A description of all covariates tested
☒	☐	A description of any assumptions or corrections, such as tests of normality and adjustment for multiple comparisons
☐	☒	A full description of the statistical parameters including central tendency (e.g. means) or other basic estimates (e.g. regression coefficient) AND variation (e.g. standard deviation) or associated estimates of uncertainty (e.g. confidence intervals)
☐	☒	For null hypothesis testing, the test statistic (e.g. *F*, *t*, *r*) with confidence intervals, effect sizes, degrees of freedom and *P* value noted<br>*Give P values as exact values whenever suitable.*
☒	☐	For Bayesian analysis, information on the choice of priors and Markov chain Monte Carlo settings
☒	☐	For hierarchical and complex designs, identification of the appropriate level for tests and full reporting of outcomes
☒	☐	Estimates of effect sizes (e.g. Cohen's *d*, Pearson's *r*), indicating how they were calculated

Our web collection on statistics for biologists contains articles on many of the points above.

Software and code

Policy information about availability of computer code

Data collection	The amino acid sequences of the MoCel12A homologous proteins were downloaded from the UniProt website (https://www.uniprot.org/).
Data analysis	SPSS 18.0, MO.Affinity Analysis, GraphPad Prism 8, MEGA 7.0 and ClustalW were used.

For manuscripts utilizing custom algorithms or software that are central to the research but not yet described in published literature, software must be made available to editors and reviewers. We strongly encourage code deposition in a community repository (e.g. GitHub). See the Nature Research guidelines for submitting code & software for further information.

Data

Policy information about availability of data

All manuscripts must include a data availability statement. This statement should provide the following information, where applicable:

- Accession codes, unique identifiers, or web links for publicly available datasets
- A list of figures that have associated raw data
- A description of any restrictions on data availability

The authors declare that all data supporting the findings of this study are available within the manuscript and the Supplementary Files or are available from the corresponding author upon request. The RNA-seq data that support the findings of this study have been deposited in the SRA database of National Center for Biotechnology Information with the accession code PRJNA707110. Mass spectrometry data that support the findings of this study have been deposited in MetaboLights with the accession code MTBLS2548. The source data underlying Figs 1, 2e-f, 2a-c, 3, 4b-g, 5a-c, 5f-g and Supplementary Figs 1b-f, 2a and 2b, 3, 4, 5a-e, 6b and 6c, 7c and 7d, 9a-c, 9e are provided as a Source Data file.

Field-specific reporting

Please select the one below that is the best fit for your research. If you are not sure, read the appropriate sections before making your selection.

- [x] Life sciences
- [ ] Behavioural & social sciences
- [ ] Ecological, evolutionary & environmental sciences

For a reference copy of the document with all sections, see nature.com/documents/nr-reporting-summary-flat.pdf

Life sciences study design

All studies must disclose on these points even when the disclosure is negative.

Sample size	The sample size was determined according to the reports in the related research subjects.
Data exclusions	No data were excluded from the analyses.
Replication	At least 3 replicates were performed and successful.
Randomization	The experiments were performed to compare between WT or Mock to the transgenic lines or treated samples, so samples allocation is not relevant to the study.
Blinding	The experiments were performed to compare between WT or Mock to the transgenic lines or treated samples, so blinding was not relevant to the study.

Reporting for specific materials, systems and methods

We require information from authors about some types of materials, experimental systems and methods used in many studies. Here, indicate whether each material, system or method listed is relevant to your study. If you are not sure if a list item applies to your research, read the appropriate section before selecting a response.

Materials & experimental systems

n/a	Involved in the study	
[ ]	[x]	Antibodies
[x]	[ ]	Eukaryotic cell lines
[x]	[ ]	Palaeontology and archaeology
[x]	[ ]	Animals and other organisms
[x]	[ ]	Human research participants
[x]	[ ]	Clinical data
[x]	[ ]	Dual use research of concern

Methods

n/a	Involved in the study	
[x]	[ ]	ChIP-seq
[x]	[ ]	Flow cytometry
[x]	[ ]	MRI-based neuroimaging

Antibodies

Antibodies used	anti-FLAG (Sigma, F3165), anti-HA(Huaxingbio, HX1820),anti-phospho-p38MAPK (Cell Signaling, 9211), anti-GFP (Transgen, HT801) and anti-Rubisco (Huaxingbio, HX1989).
Validation	anti-FLAG (1:10000, https://www.sigmaaldrich.com/), anti-phospho-p38 MAPK(1:5000, https://www.cellsignal.cn/), anti-GFP (1:5000, https://www.transgen.com.cn/), anti-HA(1:5000) and anti-Rubisco (1:5000) (http://www.huaxingbio.com/).
